# The Role of Flagellin B in *Vibrio anguillarum*-Induced Intestinal Immunity and Functional Domain Identification

**DOI:** 10.3389/fimmu.2021.774233

**Published:** 2021-11-29

**Authors:** Quanxin Gao, Shaokui Yi, Yang Li, Jinping Luo, Qianqian Xing, Xia Yang, Ming Zhao, Minghua Min, Qian Wang, Yabing Wang, Lingbo Ma, Shiming Peng

**Affiliations:** ^1^ Key Laboratory of Marine and Estuarine Fisheries, Ministry of Agriculture, East China Sea Fisheries Research Institute, Chinese Academy of Fishery Sciences, Shanghai, China; ^2^ Zhejiang Provincial Key Laboratory of Aquatic Resources, Conservation and Development, Key Laboratory of Aquatic Animal Genetic Breeding and Nutrition, Chinese Academy of Fishery Sciences, College of Life Science, Huzhou University, Huzhou, China

**Keywords:** *Vibrio anguillarum*, flagellin, *flaB*, TLR5, structure-activity relationship, protein-protein interaction

## Abstract

*Vibrio anguillarum*, an opportunistic pathogen of aquatic animals, moves using a filament comprised of polymerised flagellin proteins. Flagellins are essential virulence factors for *V. anguillarum* infection. Herein, we investigated the effects of flagellins (*flaA*, *flaB*, *flaC*, *flaD* and *flaE*) on cell apoptosis, TLR5 expression, and production of IL-8 and TNF-α. *FlaB* exhibited the strongest immunostimulation effects. To explore the functions of *flaB* in infection, we constructed a *flaB* deletion mutant using a two-step recombination method, and *in vitro* experiments showed a significant decrease in the expression of TLR5 and inflammatory cytokines compared with wild-type cells. However in the *in vivo* study, expression of inflammatory cytokines and intestinal mucosal structure showed no significant differences between groups. Additionally, *flaB* induced a significant increase in TLR5 expression based on microscopy analysis of fluorescently labelled TLR5, indicating interactions between the two proteins, which was confirmed by native PAGE and yeast two-hybrid assay. Molecular simulation of interactions between *flaB* and TLR5 was performed to identify the residues involved in binding, revealing two binding sites. Then, based on molecular dynamics simulations, we carried out thirteen site-directed mutations occurring at the amino acid sites of Q57, N83, N87, R91, D94, E122, D152, N312, R313, N320, L97, H316, I324 in binding regions of *flaB* protein by TLR5, respectively. Surface plasmon resonance (SPR) was employed to compare the affinities of *flaB* mutants for TLR5, and D152, D94, I324, N87, R313, N320 and H316 were found to mediate interactions between *flaB* and TLR5. Our comprehensive and systematic analysis of *V. anguillarum* flagellins establishes the groundwork for future design of flagellin-based vaccines.

## Introduction

*Vibrio anguillarum*, one of the most common bacteria in the marine environment, is widely distributed in coastal and estuarine seawater (1). *V. anguillarum* is a representative opportunistic bacterial pathogen that causes disease in marine fish. It causes vibriosis in both wild and farmed fish and other aquatic animals, resulting in huge losses to aquaculture (2). Therefore, *V. anguillarum* is considered a significant threat to commercial production in marine aquaculture. At least 50 species of fish can be infected with *V. anguillarum*, including pacific salmon, Atlantic salmon, rainbow trout, turbot, perch, sea carp, striped bass, codfish and Japanese flounder (3, 4). In view of the wide host range and economic losses caused by this devastating pathogen, elucidating its infection mechanism is vital for the development of control methods.

During the initial stages of pathogenesis, *V. anguillarum* uses flagellum-mediated motility to infect and colonise the surface and gut of fish hosts. Flagella are involved in both motility and virulence (5). Flagellin, the main protein of bacterial flagella, is the only known agonist of toll-like receptor 5 (TLR5), and it stimulates intestinal inflammation by activating the TLR5 signalling pathway (6, 7). Normally, this activation triggers an immunological cascade that stimulates both innate and adaptive immune responses, contributing to a reduction in bacterial multiplication in the gut. Therefore, flagellin-TLR5 interaction plays an important role in infection of pathogenic bacteria (8). However, interactions between flagellin and TLR5 in different bacteria and hosts are complex and can differ significantly, making it challenging to assess their relative contributions to pathology. Surprisingly, flagellin from a minority of bacteria cannot induce the activation of TLR5, which is critical for the survival of these bacteria at mucosal sites in hosts, and raises the intriguing possibility that flagellin receptors provided the selective force to drive the evolution of these unique subclasses of bacterial flagellins (9, 10).

To better understand bacterial flagellins, their domains, which are critical for immune activation, have been intensively investigated. Flagellin proteins contain four structural domains, D0, D1, D2 and D3, arranged in a boomerang-like structure (11). The N- and C-terminal regions that form the D0-D1 domains are highly conserved among flagellins of different bacterial species, whereas amino acid sequences of D2 and D3 show greater variability in sequence and structure (12). The D0 and D1 domains form the inner core of the filament, while the variable D2 and D3 domains form the surface. The conserved D1 domain necessary for filament assembly can be recognised by TLR5 and induces the production and release of pro- and anti-inflammatory cytokines (13). Structural and functional studies have identified many residues in the D1 domain as the interaction site with TLR5 (14), and the D0 domain also contributes to TRL5 activation (15).

Several studies have shown that *V. anguillarum flaB* and its D1 domain can activate TLR receptor pathways, and significantly upregulate tumour necrosis factor α (TNF-α) and proinflammatory cytokines (16–18). Despite increased knowledge of the structural and functional roles of flagellins in *V. anguillarum*, the molecular interactions between TLR5 and flagellins remain unclear. In this study, we examined the interaction characteristics between flagellins from *V. anguillarum* and TLR5 from silvery pomfret (*Pampus argenteus*), an economically important fish in China that is regarded a delicacy by Chinese consumers due to its favourable meat properties. Unfortunately, infectious diseases occur frequently when rearing silvery pomfret, and *V. anguillarum* is one of the main pathogens, causing huge economic losses. We speculate that flagellin-induced TLR5 activation plays an important role in the host response to infection with *V. anguillarum*. Thus, we constructed a *flaB* deletion strain and investigated the virulence of the δ*flaB* mutant *via in vivo* and *in vitro* assays. The mechanism of interaction between TLR5 and *flaB* through were also investigated, and molecular dynamics simulation and surface plasmon resonance (SPR) experiments were carried to identify the functional domains of *flaB* at the amino acid sequence level.

## Materials and Methods

### Bacterial Strains and Growth Conditions

*V. anguillarum* cells were isolated from diseased farmed fish, cultured in LB broth at 28°C, and stored in 30% glycerol at -80°C.

### Expression and Purification of Flagellins

Full-length flagellin A−E genes from *V. anguillarum* (*flaA*−*E*) were obtained by PCR using primers listed in **Table 1**. PCR products were purified from a 1.5% agarose gels and cloned into the pET28 expression vector using GeneArt Seamless Cloning and Assembly Enzyme Mix (Invitrogen). Competent *E. coli* (DE3) cells were separately transformed with pET28-*flaA*−*E* constructs, and transformants were cultured in LB medium (50 μg/mL kanamycin) at 37°C, then in SOC medium (50 μg/mL kanamycin) at 37°C for another 2−3 h to OD_600_ 0.5−0.6. IPTG was added to a final concentration of 0.5 mM to induce expression of flagellins, and bacteria were collected and resuspended in phosphate-buffered saline (PBS) with 1% Triton X-100.

**Table 1:** Sequences of primers used for cloning and expression of flagellins and TLR5.

Primer name		Sequence (5’−3’)
*FlaA*	*FlaA*-F	ACAGCAAATGGGTCGCGGATCCACCATTACAGTAAATACTAACGTC (BamHI)
	*FlaA*-R	GCAAGCTTGTCGACGGAGCTCTTACTGCAATAGTGACATTGCAG (SacI)
*FlaB*	*FlaB*-F	ACAGCAAATGGGTCGCGGATCCGCAATTAATGTAAGCACTAACGTG (BamHI)
	*FlaB*-R	GCAAGCTTGTCGACGGAGCTCTTAACCCAATAGACTAAGAGCTG (SacI)
*FlaC*	*FlaC*-F	ACAGCAAATGGGTCGCGGATCCGCGGTTAATGTAAACACTAACGTT (BamHI)
	*FlaC*-R	GCAAGCTTGTCGACGGAGCTCTTAACCAAGCAAACCAAGAGCAG (SacI)
*FlaD*	*FlaC*-F	ACAGCAAATGGGTCGCGGATCCGCAGTTAATGTAAATACCAACGTA (BamHI)
	*FlaC*-R	GCAAGCTTGTCGACGGAGCTCTTAGCCAAGTAAGCTAAGTGCCG (SacI)
*FlaE*	*FlaC*-F	ACAGCAAATGGGTCGCGGATCCGCAATTACCGTTAATACCAATGTT (BamHI)
	*FlaC*-R	GCAAGCTTGTCGACGGAGCTCCTAGCGAAGTAAGGCCAATGCTA (SacI)
TLR5	TLR5-F	ACAGCAAATGGGTCGCGGATCCACGTCGCTGGTTCTTCACTTG (BamHI)
	TLR5-R	GCAAGCTTGTCGACGGAGCTCTTAGTTTTCTTCAAGTTTGAGAAAGTG (SacI)

The meaning of the gray shadings is the restriction sites, and the restriction sites are in the brackets.

Bacteria were lysed by sonication (200−300 W) and bacterial lysates were centrifuged at 12,000 rpm for 10 min at 4°C Supernatants and precipitates were separated and analysed by sodium dodecyl sulphate-polyacrylamide gel electrophoresis (SDS-PAGE). Recombinant proteins in soluble supernatants were purified from inclusion bodies under denaturing conditions using Ni-NTA Agarose (Qiagen). Protein concentration was determined using a BCA protein assay kit according to the manufacturer’s instructions (Beyotime).

### Isolation, Culture and Stimulation of Primary Fish Intestinal Epithelial Cells (FIECs)

Fish intestinal epithelial cells (FIECs) were isolated from silvery pomfret as an *in vitro* model, and prepared and cultured according to our previous studies (19). FIECs were allowed to attach to and grow in 24-well tissue culture plates (Costar) for 48 h. Before stimulation assays, all flagellins were resuspended in DMEM medium at a concentration of 1 μg/mL. FIECs were incubated with *flaA*−*flaE* in 5% CO_2_ at 28°C After incubation for 30, 60, 120 and 150 min, the culture medium and cells were collected for ELISA, real-time PCR analysis, and assessment of apoptotic and necrotic cells (20).

### Assessment of Apoptotic and Necrotic Cells

Apoptosis and necrosis of FIECs were assessed using an Annexin V-FITC apoptosis detection kit (BD, USA). After stimulation with flagellins, cells were stained with Anexin V-fluorescein isothiocyanate (Annexin V-FITC) and propidium iodide (PI) for analysis by flow cytometry (FCM). Annexin V-FITC and PI fluorescence levels were measured at 530 nm and 585 nm emission wavelengths, respectively. The positioning of quadrants on Annexin V/PI dot plots was used to distinguish between living cells (Annexin V^-^/PI^-^), early apoptotic cells (Annexin V^+^/PI^-^), late apoptotic cells (Annexin V^+^/PI^+^) and necrotic cells (Annexin V^-^/PI^+^). Therefore, the total apoptotic proportions among cells with fluorescent Annexin were V^+^/PI^-^ and V^+^/PI^+^.

### ELISA

Protein concentration was assayed by ELISA using a Protein Assay kit (Nanjing Jiancheng Bioengineering Institute, Nanjing, China). ELISA results were quantified using an iMark Microplate Reader (Bio-Rad) at 450 nm. Absorbance was measured and protein concentration was calculated according to the relevant standard curve constructed from proteins in ELISA kits (20).

### Construction of the *flaB* Deletion Mutant Strain

Construction of the *flaB* deletion mutant strain was performed as described in our previous study (21). Briefly, the upstream homologous arm fragment (593 bp) and the downstream homologous arm fragment (545 bp) of *flaB* were amplified, and the fusion fragment (1138 bp) was obtained by overlapping PCR using the two fragments as templates. The purified PCR product was cloned into the suicide vector Plp12 and transformed into *E. coli* DH5α, yielding a positive clone of 1388 bp, which was verified by PCR and DNA sequencing. The recombinant Plp12-*flaB* plasmid was extracted and transformed into wild-type *V. anguillarum* by electro transformation. After two cycles of homologous recombination, the *flaB* deletion mutant strain was constructed and verified by PCR and DNA sequencing (1413 bp).

### Bacterial Infection

*In vivo* and *in vitro* experiments were conducted to study infection by the *δflaB* mutant. In the *in vitro* experiment, FIECs were isolated and cultured. Before stimulation of bacteria, *δflaB* mutant and wild-type strains were collected and resuspended in antibiotic-free DMEM medium at a density of 1×10^8^ CFU/mL. FIECs were coincubated with DMEM medium, *δflaB* mutant, and wild-type strains in 5% CO_2_ at 28°C for 2 h, respectively. After incubation, the culture medium and cells were collected for ELISA and real-time PCR analysis.

**Figure 1:**
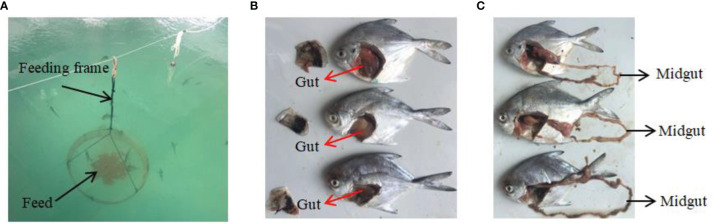
*In vivo* experiments and sampling. **(A)** Feeding device for silvery pomfret. **(B, C)** Experimental fish and sampling of intestine.

In the *in vivo* experiment, 450 silver pomfret juveniles (body weight 12−16 g) bred by artificial fertilisation were assessed. The *δflaB* mutant and wild-type strains were first cultured in LB medium for ~24 h at 28°C, centrifuged at 8000×g for 10 min, and resuspended at 1×10^10^ CFU/mL in sterile saline. Bacterial diets were prepared by gently spraying the bacterial suspension on the basal diet followed by mixing thoroughly to obtain a final cell density of 1×10^8^ CFU/mL. The bacterial feeding experiment was carried out in nine cement pools (length 6 m, width 4 m, height 1.5 m). Each pool was randomly stocked with 50 fish, and three tanks of fish were assigned to each treatment (**Figure 1**). Three fishes were sampled at 0, 12, 24 and 48 h after bacterial feeding. All fish were anaesthetised, and intestinal samples were collected for ELISA and real-time PCR analysis. At the end of the experiment, the same part of midgut of each animal was fixed in 2.5% glutaraldehyde solution (4°C) for transmission electron microscopy (TEM) analysis.

### Real-Time PCR Analysis

The methods for RNA extraction, cDNA synthesis and real-time PCR were performed according to our previous study (22).

### TEM Analysis

The method used for TEM examination was described in a previous study (23). Briefly, samples were fixed in 2.5% glutaraldehyde solution, then in 1% osmium tetroxide (O_s_O_4_) for 1 h, dehydrated in a graded series of ethyl alcohol, and embedded in resin. Ultrathin sections were placed on copper grids, stained with uranyl acetate, and observed under a TECNAI 10 TEM instrument (Philips).

### Native PAGE Analysis

The method used to obtain the extracellular region of the TLR5 protein (erTLR5) was the same as that used for expression and purification of flagellins, and primers are listed in **Table 1**. Purified *flaB* and erTLR5 proteins were incubated at 18°C for 30 min and complex formation was analysed. Native PAGE was performed using polyacrylamide gels separated at room temperature at a voltage of 200 V. After electrophoresis, gels were stained with Coomassie Brilliant Blue.

### Fluorescence Observation

Green fluorescent protein (GFP)-tagged TLR5 was used to assess the effect of *flaB* on expression of TLR5. TLR5 forward primer TCTCGAGCTCAAGCTTCGAATTCGCCACCATGACGTCGCTGGTTCTTCACTT (*Eco*RI) and reverse primer GGTACCGTCGACTGCAGAATTCGTTTTCTTCAAGTTTGAGAAAGTG (*Eco*RI) were employed for construction of erTLR5-PEGFP-N2 using GeneArt Seamless Cloning and Assembly Enzyme Mix (Invitrogen). Zebrafish embryo fibroblast cells (ZF4) were grown in DMED supplemented with 10% foetal bovine serum (FBS) and cultured in 6-well tissue culture plates (Costar) for about 24−48 h. The cell density reached 60−80% confluency prior to transfection. The erTLR5-pEGFP-N2 plasmid encoding GFP-erTLR5 was transfected into ZF4 cells using Lipo2000 (Thermo Fisher) according to the manufacturer’s instructions. The *flaB* protein was then added to 6-well plates and incubated at 28°C for 2 h. After incubation, laser scanning confocal microscopy (LSCM) was employed to observe the location and immunofluorescence expression of GFP-erTLR5.

### Yeast Two-Hybrid Analysis

The primers listed in **Table 2** were used to amplify the sequences of *flaB* (1128 bp) and *TLR5* (1770 bp). The purified *flaB* and *TLR5* sequences were ligated into pGADT7 and pGBKT7 vectors, respectively, and recombinant pGADT7-*flaB* and pGBKT7-erTLR5 vectors were simultaneously transformed into yeast strain AH109. Transformants were grown on synthetic dropout medium lacking amino acids leucine and tryptophan (SD/–Leu/–Trp), synthetic dropout medium lacking adenine and amino acids histidine, leucine and tryptophan (SD/–Ade/–His/–Leu/–Trp) and SD/–Ade/–His/–Leu/–Trp/X-α-Gal (40 μg/mL X-α-Gal) on agar plates at 30°C for 3−5 days to assess growth.

**Table 2:** Sequences of primers used for yeast two-hybrid analysis.

Primer name		Sequence (5’−3’)
*FlaB*	*FlaB*-F	GCCATGGAGGCCAGTGAATTCATGGCCATTAACGTTT (EcoRI)
	*FlaB*-R	TGCAGCTCGAGCTCGATGGATCCACCCAACAAAGACAAA (BamHI)
erTLR5	erTLR5-F	GCATATGGCCATGGAGGCCGAATTCATGACGTCGCTGGTTCTTCACTTG (EcoRI)
	erTLR5-R	GCGGCCGCTGCAGGTCGACGGATCCTCAGTTTTCTTCAAGTTTGAGAAAGTG (BamHI)

The meaning of the gray shadings is the restriction sites, and the restriction sites are in the brackets.

### Modelling of *flaB* Interaction With TLR5 Using PROCHECK and Rosetta

The 3J0A and 3K8W proteins served as templates for homology modelling of TLR5 and *flaB* proteins, respectively, using Modeller v9.19. The two models were then subjected to molecular mechanics optimisation, and PROCHECK was used to evaluate the optimised protein models. Analysis of the Ramachandran plot showed that 99.1% and 99.3% of non-glycine residues of TLR5 and *flaB* were in the ‘most favoured or allowed’ regions. Finally, we used Rosetta to carry out *flaB*-TLR5 docking.

### *In Vitro* Mutagenesis

The amino acid sites selected for mutation are shown in **Table 3**. KOD FX DNA polymerase (Toyobo) was applied to construct the mutated genes from the wild-type pET28-*flaB* template *via* thermal cycling at 95°C for 3 min followed by 25 cycles at 95°C for 15 s, 62°C for 15 s, 68°C for 1 min, and a final extension at 72°C for 5 min. FastDigest *Dpn*I (Thermo Fisher) was added to the PCR product and incubated in a 37°C water bath for 2 h. The mixture was purified using a PCR Clean-Up Kit and transformed into DMT competent cells. Transformants were selected for sequencing using T7 promoter and T7 terminator primers.

**Table 3:** Sequences of primers used for site-directed mutagenesis of *flaB*.

FlaB	Abbreviation	Amino acid mutation	Sequence (mutation sites are shaded grey)
Q57	F1	Gln—Ala	F: 5’ CGTCAGCAAGCCGTGGCTTAG 3’
			R: 5’ GCCACGGCTTGCTGACGTTAAAC 3’
N83	F2	Asn—Ala	F: 5’ GGTGCAATGGCTGAAACCACCAA 3’
			R: 5’ GATATTGGTGGTTTCAGCCATTG 3’
N87	F3	Asn—Ala	F: 5’ CCACCGCTATCTTGCAACGTATG 3’
			R: 5’ CGTTGCAAGATAGCGGTGGTTTC 3’
R91	F4	Arg—Ala	F: 5’ CTTGCAAGCTATGCGAGATCTTTC 3’
			R: 5’ GATCTCGCATAGCTTGCAAGATA 3’
D94	F5	Asp—Ala	F: 5’ CGTATGCGAGCTCTTTCTTTG 3’
			R: 5’ GCAAAGAAAGAGCTCGCATA 3’
E122	F6	Glu—Ala	F: 5’ GATGCATTAAACCGTATTGCTGAA 3’
			R: 5’ GCAATACGGTTTAATGCATCATTG 3’
D152	F7	Asp—Ala	F: 5’ GGTGCCGCCTCTGGTGAA 3’
			R: 5’ CTTCACCAGAGGCGGCACCAAT 3’
N312	F8	Asn—Ala	F: 5’ CCAGGCCCGTTTCGGTCATG 3’
			R: 5’ GACCGAAACGGGCCTGGAATG 3’
R313	F9	Arg—Ala	F: 5’ CCAGAACGCTTTCGGTCAT 3’
			R: 5’ GACCGAAAGCGTTCTGGAATG 3’
N320	F11	Asn—Ala	F: 5’ GCCATCAGTGCCCTTGATAAC 3’
			R: 5’ CAAGGGCACTGATGGCATGA 3’
L97	F12	Leu—Asn	F: 5’ GAGATCTTTCTAACCAATCAGCA 3’
			R: 5’ CGTTTGCTGATTGGTTAGAAAG 3’
H316	F13	His—Ala	F: 5’ CGTTTCGGTGCTGCCATCAGTA 3’
			R: 5’ GGTTACTGATGGCAGCACCGAAA 3’
I324	F14	Ile—Asn	F: 5’ GATAACAACAACGAGAACGTCAA 3’
			R: 5’ CGTTCTCGTTGTTGTTATCAAGGT 3’

### Surface Plasmon Resonance (SPR) Analysis

Prokaryotic expression and purification of mutant *flaB* was performed as described above. The TLR5 gene was inserted into the pGEX-6p-1 vector, and after its transformation into BL21(DE3) strain and induction by IPTG, recombinant protein was expressed in BL21 cells and purified using glutathione S-transferase (GST) affinity chromatography. The GST-His*10-TEV-TLR5 fusion protein was digested by tobacco etch virus (TEV) protease to remove the GST tag, and purified TLR5 protein was obtained.

Analysis of ligand binding kinetics was performed on a BIAcore S200 SPR instrument (GE Healthcare, USA) according to methods reported previously (24). In brief, TLR5 protein was immobilised onto the SA sensor chip surface. *FlaB* mutant proteins were injected into wells at a flow rate of 30 μL/min in PBS-P running buffer. After 5 min of dissociation, TLR5 and bound analytes were removed by a 60 s wash with 10 mmol/mL glycine-HCl at a flow rate of 30 mL/min. After subtraction of appropriate controls, data were analysed by fitting to a 1:1 Langmuir binding model using the BIAcore S200 evaluation software.

## Results

### *In Vitro* Analysis of Flagellins in Infection

**Figure 2:**
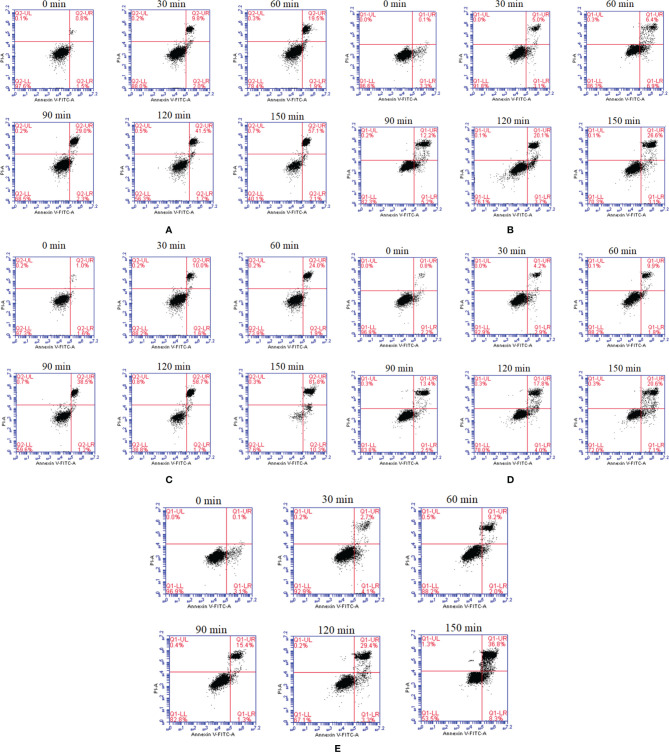
*In vitro* dynamic monitoring of FIECs infected with flagellins by flow cytometry. The *flaA* **(A)**, *flaB* **(B)**, *flaC* **(C)**, *flaD* **(D)** and *flaE* **(E)** genes were separately added into FIECs. At definite time intervals of 30, 60, 120 and 150 min, cells were collected, stained with Annexin V-fluorescein and PI, and analysed by flow cytometry. LL, living cells; LR, early apoptotic cells; UR, late apoptotic cells; UL, necrotic cells.

To gain insight into the functions of flagellins, we expressed and purified the *flaA−E* and investigated their effects on cell apoptosis and necrosis. All five flagellins were shown to be capable of inducing apoptosis and necrosis in FICEs in a time-dependent manner through the observation of cells at 0, 30, 60, 120 and 150 min (**Figure 2**). After the final incubation at 28°C for 150 min, the *flaB* markedly increased early (Annexin V+/P-) and late (Annexin V+/PI+) apoptosis rates to 10.3 and 81.8, respectively. Among the flagellins, *flaB* was ranked most effective at inducing cell apoptosis (92.1%), followed by *flaA* and *flaE* (59.2% and 45.1%, respectively). Cell apoptosis induced by *flaC* and *flaD* showed the same trend, albeit with lower rates of 29.7% and 27.7%, respectively. Overall, here was a notable difference in the sensitivity of FICEs to different flagellins, and *flaB* was the most capable of inducing apoptosis.

**Figure 3:**
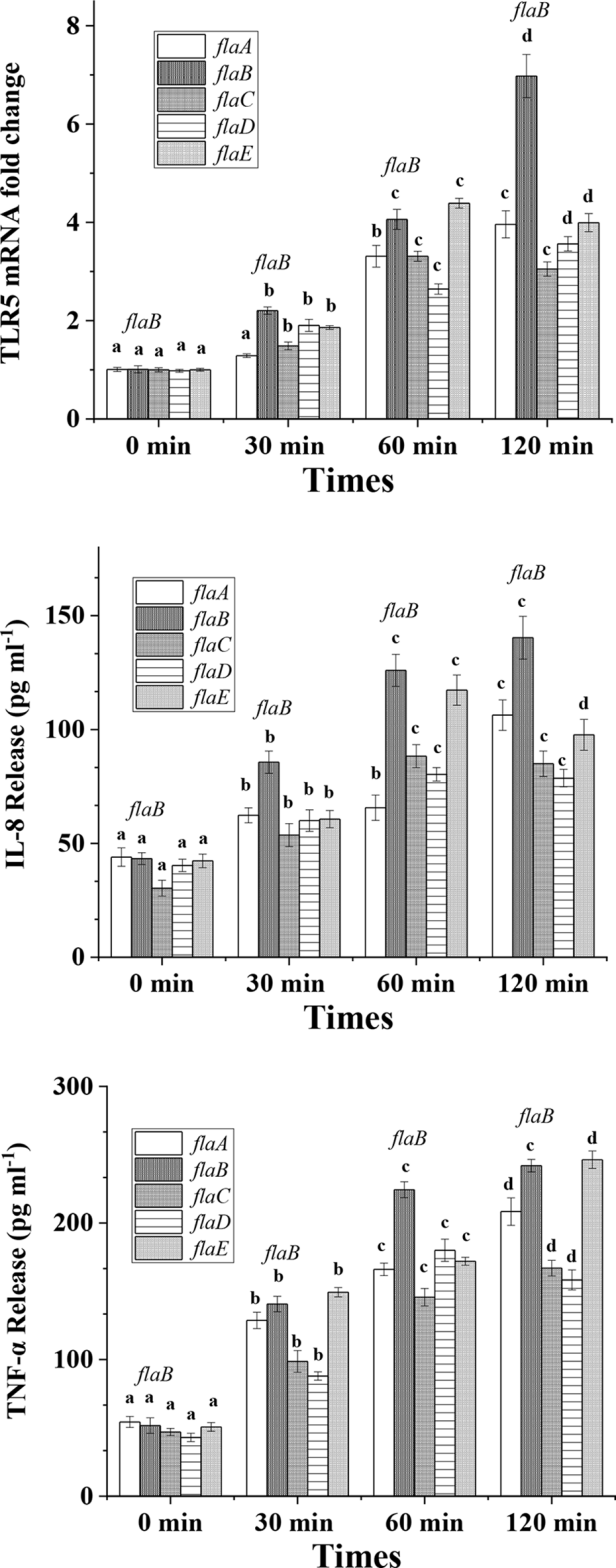
TLR5, IL-8 and TNF-α expression in cultured FIECs after addition of flagellins. Cells were exposed to *flaA*, *flaB*, *flaC*, *flaD* and *flaE* for 30, 60 and 120 min. After incubation, cells were harvested for quantitative real-time PCR to determine TLR5 expression levels. Supernatants were collected and TNF-α and IL-8 concentrations in the supernatant were measured by ELISA. Results are means ± standard errors of the means for three independent experiments. Different lowercase letters indicate significant differences between different time points within the same treatment.

Following cell apoptosis analysis, expression of TLR5, IL-8 and TNF-α was measured. As shown in **Figure 3**, all five flagellins increased the expression of TLR5 significantly and in a time-dependent manner. After incubation for 120 min, *flaB* -induced TLR5 expression was more significant than that of other flagellins (*p <*0.05). Similar to TLR5 gene expression, secretion levels of IL-8 and TNF-α proteins from FICEs were increased significantly after infection with flagellins (*p <*0.05). Significantly higher production of IL-8 was observed in the *flaB* treatment group than the other flagellin groups. Unlike other groups, IL-8 expression in the *flaE* treatment group increased significantly at 30 min, peaked at 60 min, and decreased significantly at 120 min (*p <*0.05). Except for *flaD*, TNF-α expression varied in a time-dependent manner after treatment with flagellins; *flaB* and *flaE* had the strongest stimulatory effects on the expression of TNF-α (*p <*0.05), and there was no significant difference between these two groups.

### *In Vivo* Analysis of Infection

**Figure 4:**
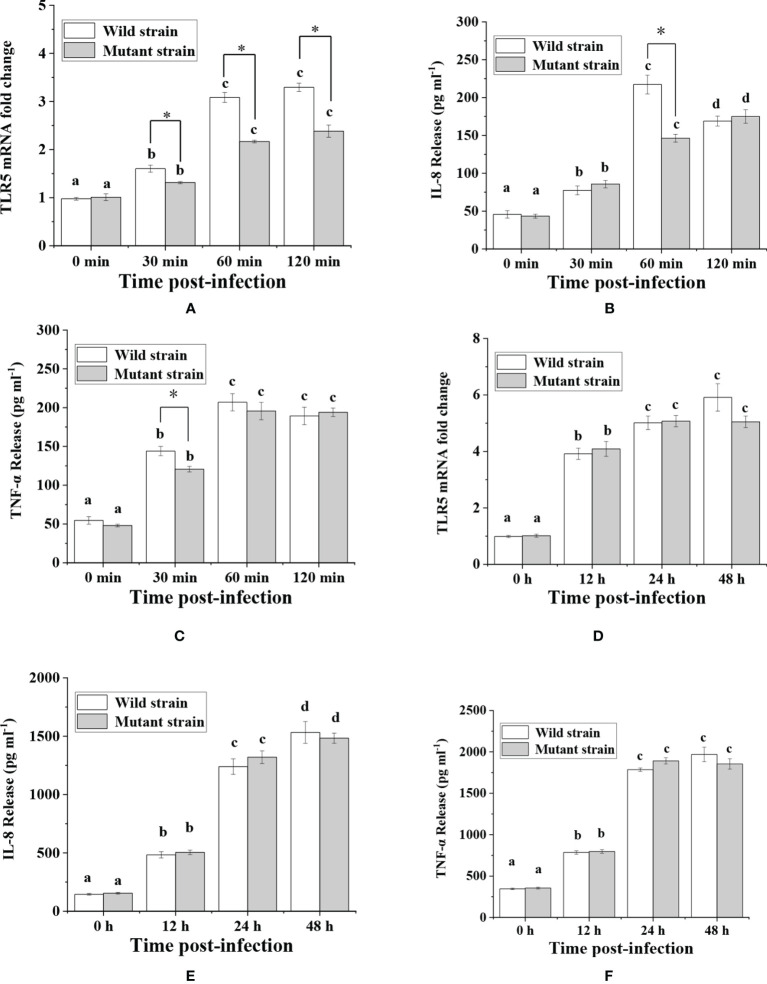
Analysis of TLR5, IL-8 and TNF-α expression in response to V*. anguillarum* and its mutant challenge. Time-lag experiments were performed to explore *in vitro* **(A–C)** and *in vivo* **(D–F)** infection with bacteria. The production of IL-8 and TNF-α proteins was measured by ELISA, and mRNA expression levels of TLR5 were measured by real-time PCR. Different lowercase letters indicate significant differences between different time points within the same treatment. * indicate significant differences between the two groups at the same time point.

Among the five flagellins, *flaB* was the most capable of inducing TLR5-meditated inflammatory responses, hence we knocked out the *flaB* gene in *V. anguillarum* and tested the infection ability of the mutant to explore the function and mechanism of *flaB.* A time course of TLR5 expression was performed by real-time PCR in FICEs after treatment with the *flaB* mutant. As shown in **Figure 4**, significantly lower expression of TLR5 was observed in the mutant group in comparison with the wild-type group at all time points. However, in the *in vivo* experiment, only at 48 h of exposure to bacteria were TLR5 mRNA expression levels in the mutant group lower than in the wild-type group; at other time points levels were comparable in the two groups.

**Figure 5:**
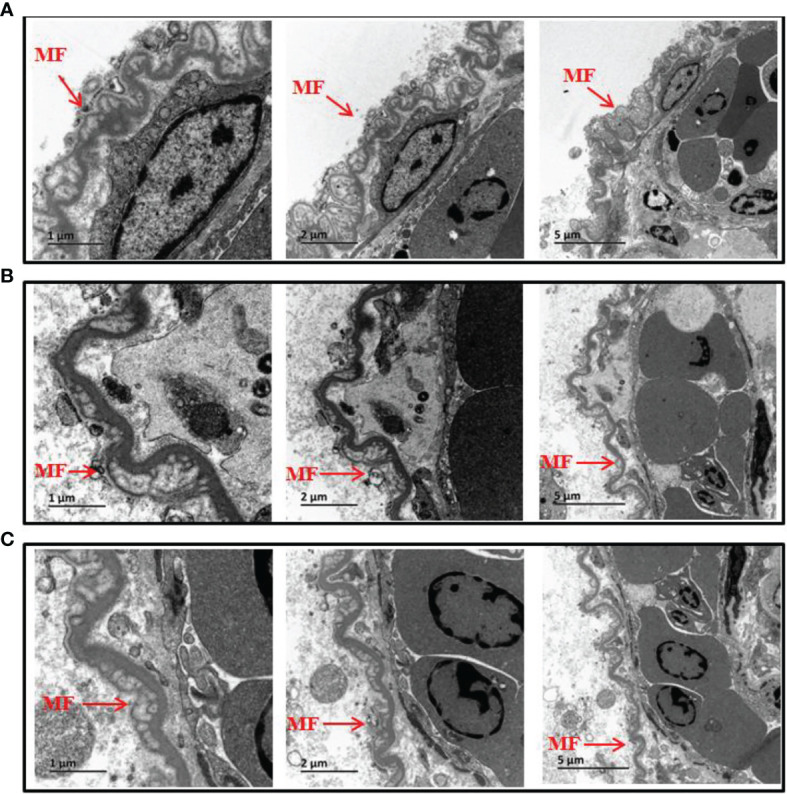
Electron microscopic structure of the intestine epithelium of silver pomfret juveniles fed with *δflaB* mutant and wild-type strains. **(A)** Controls. **(B)** Wild-type treatment. **(C)** *δflaB* mutant treatment. MF, mucosal folds. Arrows indicate the epithelium layer.

Following analysis of TLR5 expression, production of IL-8 and TNF-α was evaluated (**Figure 4**). The results showed that expression of IL-8 at 60 min and TNF-α at 30 min was significantly lower in the mutant group than in the wild*-*type group in *in vitro* experiments. However, in *in vivo* experiments there was no significant difference in IL-8 and TNF-α protein abundance between the two groups. The reason for the inconsistent *in vivo* and *in vitro* results might be because the fish gut microbiome is a complex ecosystem that can mediate interaction of the fish host with *V. anguillarum*. Therefore, we conducted TEM examination of intestinal mucosal samples. As shown in **Figure 5**, the intestinal mucosa in the control group remained intact, the epithelium was normal, and the columnar epithelium in the mucosa had an ordered arrangement. However, in wild-type and mutant *V. anguillarum* treatment groups, microvilli on the surface of epithelial cells of the intestine were sparse or distributed irregularly, the mucosal folds were flattened, and the mucosa layer was thin. Overall, there was little difference between the two treatment groups in terms of changes in the intestinal mucosal structure.

### Native PAGE and Size Exclusion Chromatography Analyses

**Figure 6:**
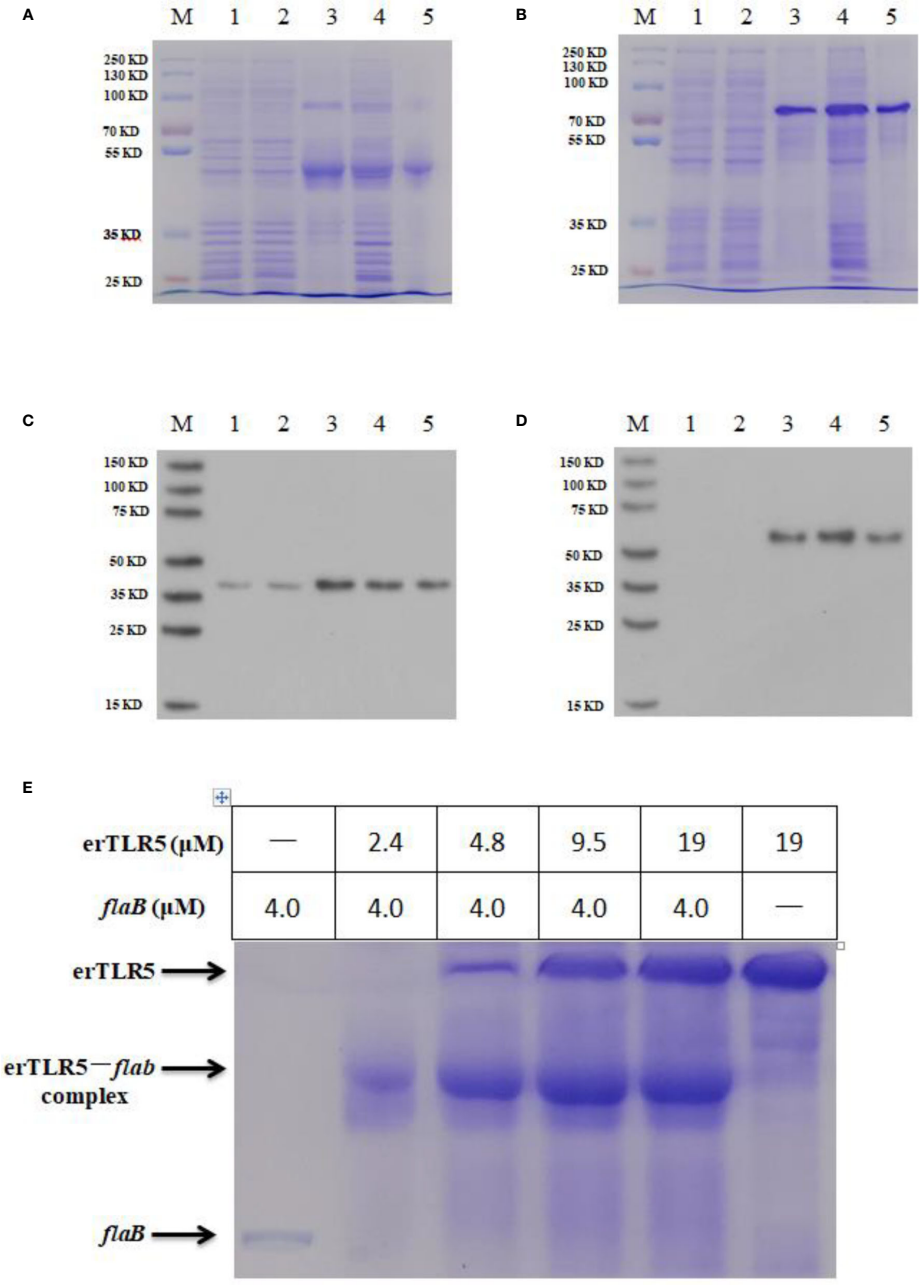
Native PAGE analysis of the direct interaction between *flaB* and erTLR5. *FlaB* **(A)** and erTLR5 **(B)** production was assessed by SDS-PAGE. *FlaB* **(C)** and erTLR5 **(D)** proteins were further validated by western blotting. Lane M, markers; lane 1, empty plasmid control; lane 2, no IPTG control; lane 3, inclusion bodies; lane 4, supernatant; lane 5, purified protein. **(E)** Native PAGE (upper panel) and size exclusion chromatography (lower panel) reveal the formation of a 1:1 erTLR5-*flaB* heterodimer.

To confirm that *flaB* -mediated TLR5 activation occurs through direct interaction between *flaB* and erTLR5, the formation of the *flaB* -erTLR5 complex was monitored by native PAGE. As shown in **Figures 6A, B**, *flaB* and erTLR5 proteins were expressed and purified, and further validated by western blotting (**Figures 6C, D**). In the native PAGE analysis, *flaB* altered erTLR5 mobility, and the mobility shift was complete at a molar ratio of 1:1 (**Figure 6E**). These results imply a relatively strong interaction between *flaB* and erTLR5.

### Fluorescence Observations

**Figure 7:**
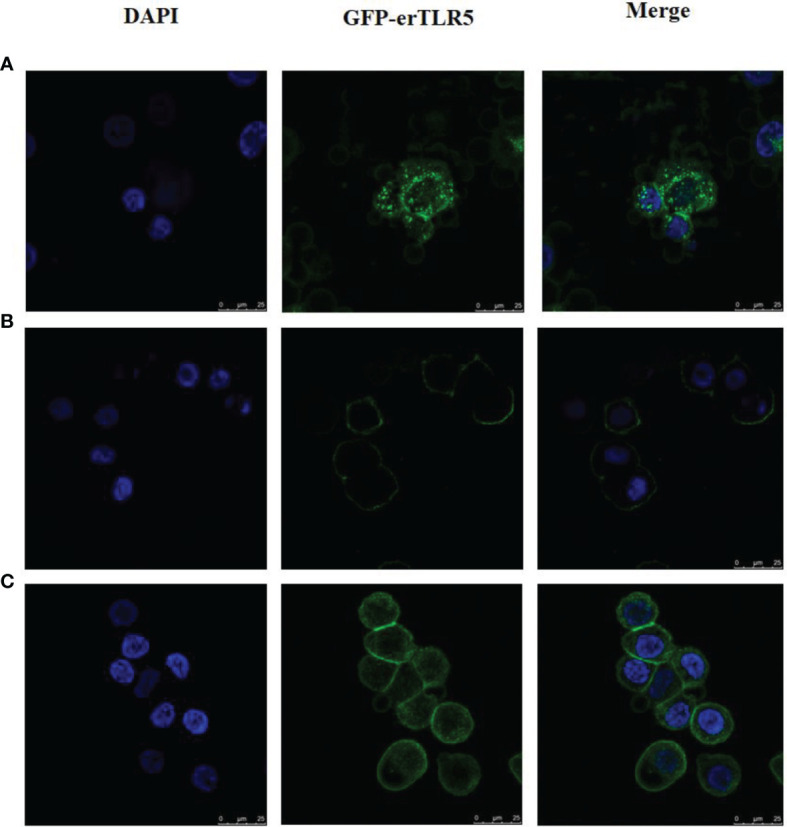
Observation and analysis of enhanced GFP-erTLR5 expression induced by *flaB* in ZF4 cells using LSCM. **(A)** Transfection of empty plasmid (pEGFP-N2). **(B)** Transfection of erTLR5-pEGFP-N2. **(C)** *FlaB* treatment. Left, staining with DAPI; middle, GFP-erTLR5; right, merge of the two images.

In order to observe the *flaB* -induced expression of TLR5 protein directly, GFP was used to label erTLR5. As shown in **Figure 7A**, GFP was expressed in the cytoplasm in the empty plasmid transfection group. In the erTLR5-pEGFP-N2 transfection group, GFP fluorescence was observed on the cell surface of ZF4 cells (**Figure 7B**), indicating that TLR5 protein was efficiently transported to the plasma membrane. Because TLR5 is a Type I membrane protein, GFP-erTLR5 proteins can be transported to the cell membrane, consistent with our observations. Furthermore, our results showed that treatment with *flaB* induced the expression of GFP-erTLR5 protein (**Figure 7C**), which further demonstrated an interaction between *flaB* and TLR5.

### Yeast Two-Hybrid Analysis

**Figure 8:**
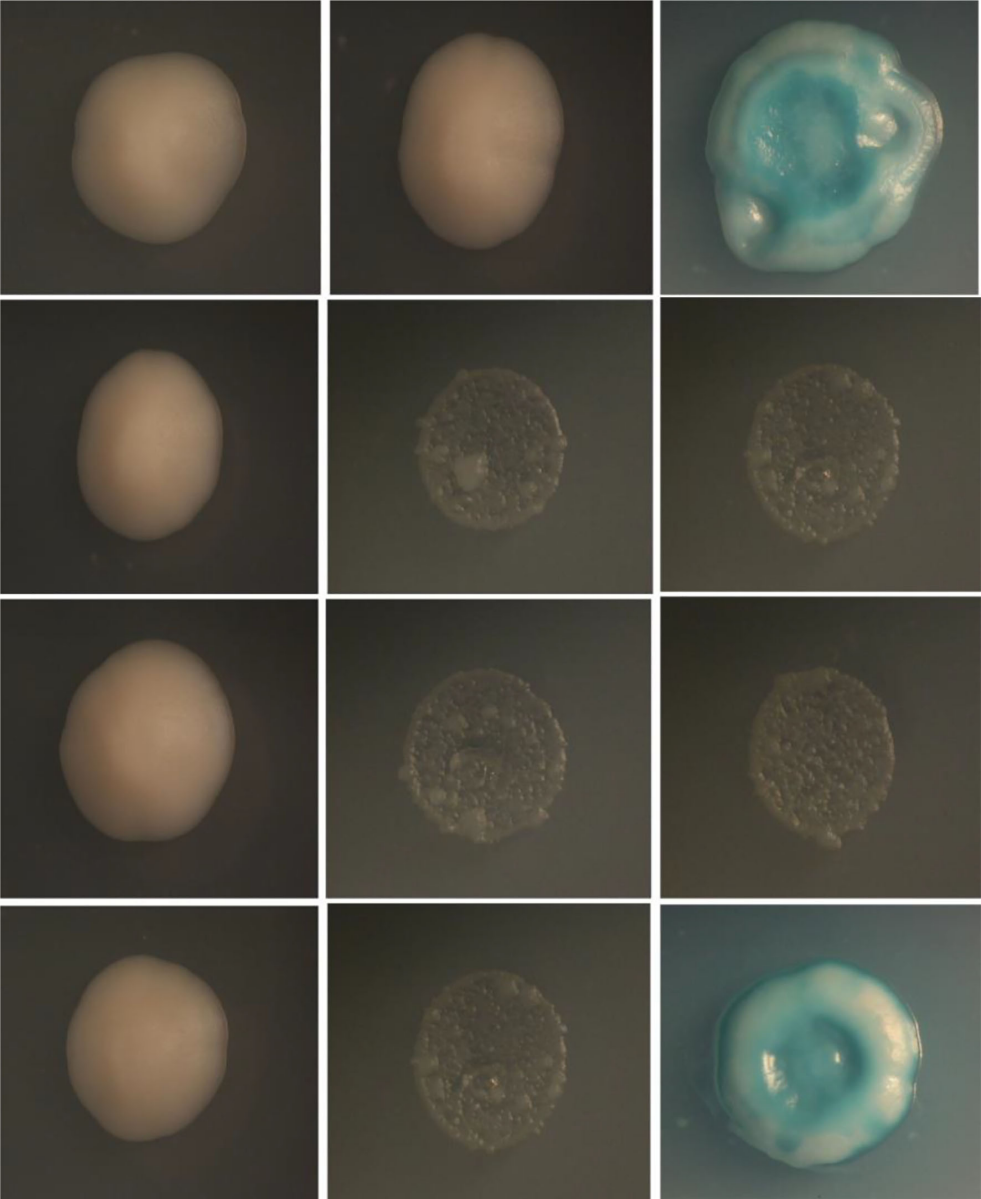
Yeast-two-hybrid screening for interaction between *flaB* and erTLR5. First column, SD-Leu-Trp medium; second column, SD/–Ade/–His/–Leu/–Trp medium; third column, SD/–Ade/–His/–Leu/–Trp/X-α-Gal medium. Fist line, positive control; second line, negative control; third line, self-activated control; fourth line, experimental group.

Yeast two-hybrid screening methods are an effective means for the detection of protein-protein interactions. Therefore, yeast two-hybrid analysis was conducted to determine if the TLR5 protein interacts directly with *flaB*. As shown in **Figure 8**, yeast transfected with pGADT7-*flaB* and pGBKT7-erTLR5 vectors could be grown on SD/–Leu/–Trp plates, indicating the successful co-transformation of the AH109 yeast strain. Additionally, growth on SD/–Ade/–His/–Leu/–Trp and SD/–Ade/–His/–Leu/–Trp/X-α-Gal medium plates (blue clones) indicated a direct interaction between *flaB* and erTLR5 (**Figure 8**).

### The Binding Interface of the *flaB* -TLR5 Complex

We docked *flaB* and TLR5 proteins, and took the highest I_sc (interface score) as representative of binding between TLR5 and *flaB*. I_sc can be used to describe the strength of the interaction between two proteins, and I_sc generally ranges from -2 to -10. Our results showed that the I_sc of the final docking result was -9.561, which confirmed that the TLR5- *flaB* complex was compatible in terms of shape and energy.

**Figure 9:**
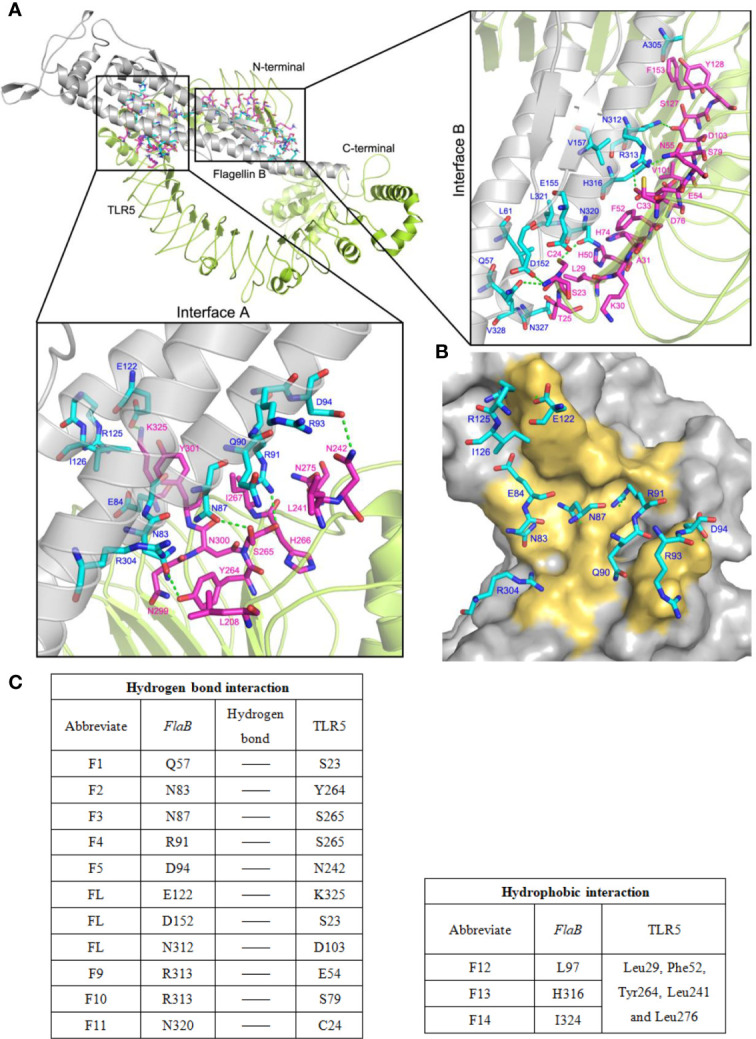
The binding model of *flaB* and TLR5 protein docking. **(A)** TLR5 is shown in cyan ribbon representation and *flaB* is shown in grey ribbon representation. Amino acid residues involved in the interaction between TLR5 and *flaB* are shown in purple and blue ribbons, respectively. The green dotted line represents hydrogen bonding between the two proteins. **(B)** The TLR5 surface (grey). Amino acid residues of *flaB* are colored blue, and amino acids of TLR5 that interact with *flaB* are colored yellow. **(C)** Amino acids involved in protein interactions.

As shown in **Figure 9**, we analysed hydrogen bonds between the binding sites of TLR5 and *flaB*. The binding area includes two main regions (Interface A and Interface B) located in the middle part and N-terminus of the TLR5 protein structure, respectively. The amino acid residues involved in binding between *flaB* and TLR5 are shown in **Table 3**. Most of the amino acids that interact with each other can do so *via* hydrogen bonds, indicating that hydrogen bonds play important roles in TLR5 recognition of *flaB*. In addition, we speculate that hydrophobic amino acids on the interface may further enhance the interactions between *flaB* and TLR5.

In order to better understand the *flaB* -TLR5 interaction, the binding interfaces and interacting amino acids in Interface A and Interface B are shown in **Figure 9**. The amino acids from *flaB* and TLR5 interact *via* numerous hydrogen bonds and hydrophobic interactions (**Figure 9C**), resulting in a strong affinity between the two proteins. According to the results of protein docking, it can be inferred that the mechanism of *flaB* - induced activation of TLR5 may involve *flaB* binding to TLR5, which leads to TLR dimerisation and conformational changes, resulting in signal transduction.

### Site-Directed Mutagenesis and Expression and Purification of *flaB* Mutant Proteins and TLR5

**Figure 10:**
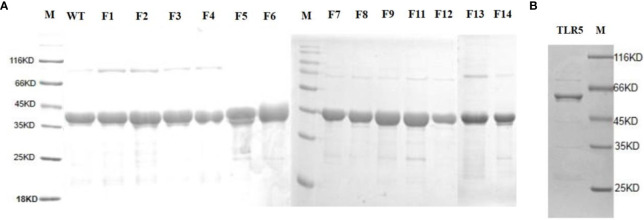
SDS-PAGE analysis of TLR5 and *flaB* mutant proteins. Lane M: protein molecular standard (kDa). **(A)** *FlaB* mutant proteins. **(B)** TLR5 protein. Lane M, protein molecular standards (kDa).

To identify the predicted functional domains in *flaB*, we performed site-directed mutagenesis and SPR experiments. Firstly, we performed site-directed mutagenesis of amino acids predicted to be involved in TLR5- *flaB* interactions. The TLR5 and *flaB* mutant proteins were successfully expressed and purified using a prokaryotic expression system. Distinct bands with molecular masses of ~39.5 kDa (**Figure 10A**) and ~64.4 kDa (**Figure 10B**) were observed, consistent with the predicted molecular masses of *flaB* and TLR5.

**Figure 11:**
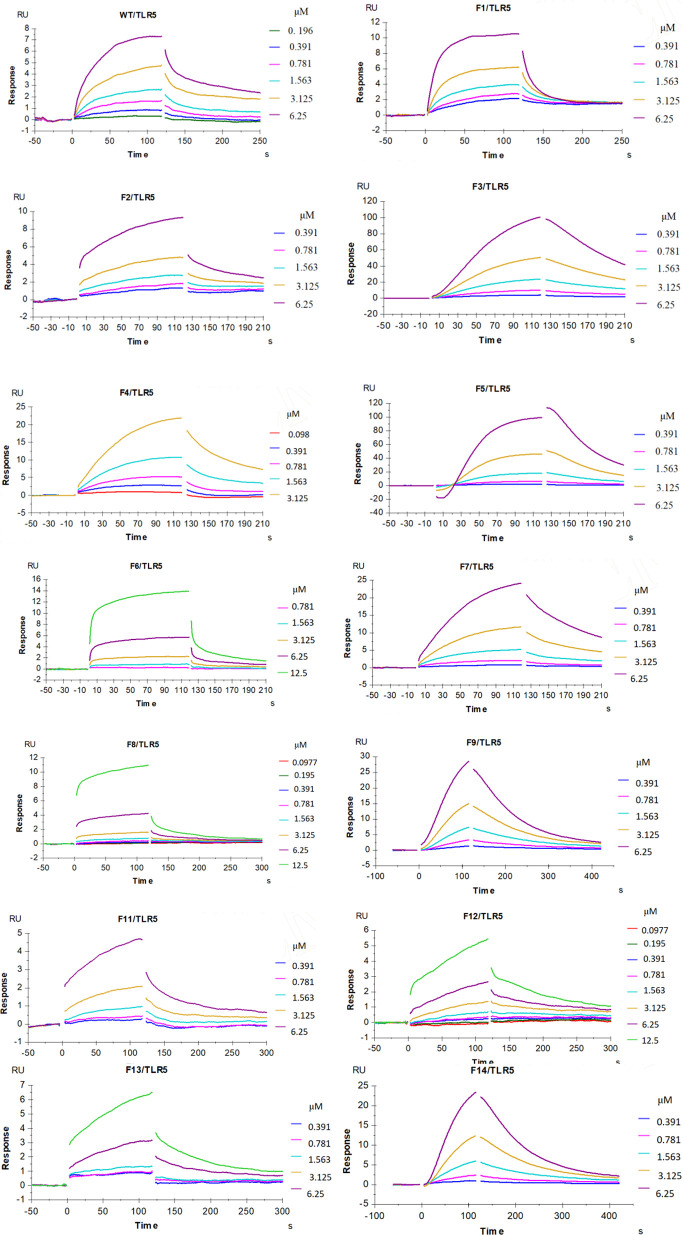
Influence of *flaB* gene site mutation upon binding to TLR5. Multiple concentrations of the indicated *flaB* mutant and wild-type proteins were passed over TLR5 immobilised on the surface of the SA sensor chip. The binding response is shown as response units (RU) and was assessed at a flow rate of 30 µL/min at 25°C.

Our enhanced SPR assay was then applied to assess the impact of the various *flaB* profiles (F1−F14 mutants) upon binding to TLR5. As shown in **Figure 11** and **Table 4**, Kd values of F1, F2, F4 and F12 decreased, while Kd values of the other *flaB* mutants increased, compared with wild-type controls. Kd values of F7, F5, F14, F3, F9, F11 and F13 increased by 21.8-, 14.1-, 8.3-, 7.9-, 4.4-, 3.9- and 2.2-fold respectively compared with controls, indicating that amino acids D152, D94, I324, N87, R313, N320 and H316 play key roles in the spatial interaction between *flaB* and TLR5.

**Table 4:** Calculated dissociation constant (Kd) values *flaB* mutants binding to TLR5 protein inferred by SPR.

FLAB	Ligand/receptor	Dissociation constant(Kd, μM)
WT	WT/TLR5	3.283
F1	F1/TLR5	0.4314
F2	F2/TLR5	0.4472
F3	F3/TLR5	26.23
F4	F4/TLR5	2.571
F5	F5/TLR5	46.34
F6	F6TLR5	4.403
F7	F7/TLR5	71.68
F8	F8/TLR5	3.796
F9	F9/TLR5	14.76
F11	F11/TLR5	12.96
F12	F12/TLR5	2.411
F13	F13/TLR5	7.29
F14	F14/TLR5	27.48

## Discussion

*V. anguillarum* is one of the most serious pathogenic bacteria affecting global aquaculture of various fish species. Due to its high morbidity and mortality rates, a substantial amount of research has been carried out to elucidate the virulence mechanisms of this pathogen, and to develop rapid detection techniques and effective disease-prevention strategies (25). It is well known that the flagella of pathogens are important for both bacterial motility and virulence. Furthermore, flagella are novel and effective adjuvants for vaccines (12, 26). However, to our knowledge, few studies have focused on the flagella of *V. anguillarum*. In the present study, constructs of flagellin gene deletion mutants revealed the roles of flagellin proteins in *V. anguillarum* infection. Additionally, interactions between flagellin proteins and TLR5 were comprehensively explored at the protein structure level.

Importantly, flagellins are usually species-specific (27), and there are significant differences in the immune properties of flagellins from different bacterial sources (28). Even from the same bacteria, the immunogenicities of different flagellins can differ markedly (29). Flagellins do not contribute equally to the virulence of pathogenic bacteria. Thus, in order to obtain a clearer picture of the roles of flagellins in *V. anguillarum*, their immune characteristics should be thoroughly investigated. In the present study, *in vitro* experiments showed that *flaB* induced the strongest immune response among the flagellins. Studies by Gonzalez-Stegmaier et al. (2019 and 2021) reported similar findings (16, 30). However, these previous studies did not delve further into the immune function mechanisms of *flaB*, hence further research is required to deepen our understanding in this area.

Knocking out flagellin genes can reduce bacterial pathogenicity, but it may also promote infection (31). Although flagellins contribute to intestinal inflammation by activating TLR5, the absence of flagellins in pathogenic bacteria can assist their ability to escape from the animal tract by avoiding triggering the proinflammatory response during invasion, thus favouring the development of systemic infection (32). Our *in vitro* experiments showed that deletion of *flaB* significantly decreased *V. anguillarum*-induced expression of TLR5, IL-8 and TNF-α. It has been reported that mutants of some flagellins have little or no effect, and only by deletion of the most influential components can bacterial infectivity be reduced (29). Based on our current experimental data and other studies, we can conclude that *flaB* is an important virulence factor. However, *in vivo* experiments showed that the *flaB* mutant displayed similar immune characteristics to the wild-type strain. We speculate that the inconsistency between *in vivo* and *in vitro* results might be due to the complexity of fish gut microbes. A large number of diverse microorganisms live in the gut of fish and other animals, and these microorganisms can work together with the host to fight against the invasion of pathogenic bacteria to maintain the stability of the living environment (33). Thus, commensal microbes may help the fish host alleviate immune stimulation caused by *flaB* during infection with *V. anguillarum*.

*FlaB*-induced TLR5 expression was visualised directly using fluorescent labelling, which confirmed that *flaB* induced TLR5 activation. Additionally, for the first time, we confirmed the interaction effects between *flaB* and TLR5 using native PAGE and yeast two-hybrid analysis. It is almost certain that *flaB* binds directly to TLR5 expressed on cultured cells, which consequently induces cytokine expression. Many studies have proved that TLR5 recognises flagellins *via* the extracellular domain, which leads to the expression of a variety of genes. However, due to variation in the sequences and domains of flagellins, flagellins from diverse bacterial species use different TLR5 recognition mechanisms (34). For activation, the receptor must undergo a ligand-induced conformational change (27). In the present study, we aimed to identify the specific ligand-binding sites on the surface of ligand-receptor complexes. Through a detailed examination of our refined docking model and its experimental validation using point mutants, we identified *flaB* residues at two different interfaces that are important for flagellin recognition, and are therefore candidates for participation in an extended TLR5 interaction site.

Successful vaccine preparations have to properly activate immune cells to induce a long-term memory protective immunity. Together with antigens of pathogen, adjuvants strongly contribute to the effectiveness of a vaccine. A number of studies have clearly demonstrated that that flagellin is a strong candidate for an engineered vaccine scaffold as it is known to provide adjuvant activity through its TLR5 and inflammasome activation (35, 36). So far, flagellin has been developed as an important versatile adjuvant applicable to wide spectrum of vaccines and immunotherapies adjuvant with great success (37, 38). It is generally known that *V. anguillarum* is a highly pathogenic bacterium of aquatic species worldwide, hence its flagellins could be actively explored as vaccine adjuvants and carriers. Compared to other flagellins, *flaB* has the strongest immunostimulatory activity, thus *flaB* is recommended for adjuvanted vaccines in order to strengthen the efficacy of the vaccine. The *flaB* adjuvants might allow more effective flagellin-based vaccines to enter clinical trials.

It is widely accepted that as an adjuvant, flagellin can help induce more potent antigen-specific immune response. However, if the functional domains of flagellins are disrupted during vaccine adjuvant production, their immunity effects may be lowered or lost (39). Therefore, structure-guided fusion-protein design using flagellin as vaccine adjuvant has been one of the research hotspots in the world scope (36). That makes it possible to avoid destroying the functional area of flagellin in the process of vaccine preparation, so as to ensure the efficacy of the vaccine (40). Therefore, the study of functional structure of *flaB* has important practical significance. In this study, according to the results of molecular simulations, SPR assays were further performed to determine the binding affinity between TLR5 and *flaB* mutant proteins generated by site-directed mutagenesis, and we identified several key amino acid residues that mediate binding of *flaB* to TLR5, which might be important for exploitation and utilization of flagellins from *V. anguillarum* (41). In order to effectively prevent and control *V. anguillarum*, development of effective vaccines is paramount, and our current research on the functional domains of flagellins will contribute toward the development of innovative vaccines against *V. anguillarum*.

In summary, we present the first comprehensive and in-depth study of flagellins in *V. anguillarum.* We performed flow cytometry, PCR and ELISA to compare the immunostimulatory effects of different flagellins in *V. anguillarum*, and *flaB* displayed the strongest ability to induce apoptosis and inflammatory responses. To further dissect the functions of *flaB* in infection, we constructed a *flaB* deletion mutant using a two-step recombination technique and found that this mutant exhibited dramatically decreased virulence in FICEs in *in vitro* experiments. Through native PAGE, yeast two-hybrid and fluorescence analyses, we further confirmed the physical interaction between *flaB* and TLR5. Finally, we analysed the functional domains and key amino acids, and revealed the roles of these amino acids in interactions between *flaB* and TLR5 using site-directed mutagenesis and SPR analyses. Our results help to reveal the properties of bacterial flagellins, and provide a basis for development of vaccines.

## Data Availability Statement

The original contributions presented in the study are included in the article/supplementary material. Further inquiries can be directed to the corresponding author.

## Ethics Statement

The animal study was reviewed and approved by Chinese Academy of Fishery Sciences.

## Author Contributions

QG, SY, and YL participated in the design of experiments, analyzed verification results and wrote the manuscript. SP, JL, and QX assisted in laboratory work. MZ, YW, QW, and MM were involved in sampling and mRNA extraction. LM and XY designed and supervised the work, plus they edited the paper. All authors contributed to the article and approved the submitted version.

## Funding

This work was supported by grants from the Natural Science Foundation of Shanghai (No.19ZR1470000), National Natural Science Foundation of China (31772870); the Central Nonprofit Basic Scientific Research Project for the Scientific Research Institutes of China (2420-2019).

## Conflict of Interest

The authors declare that the research was conducted in the absence of any commercial or financial relationships that could be construed as a potential conflict of interest.

## Publisher’s Note

All claims expressed in this article are solely those of the authors and do not necessarily represent those of their affiliated organizations, or those of the publisher, the editors and the reviewers. Any product that may be evaluated in this article, or claim that may be made by its manufacturer, is not guaranteed or endorsed by the publisher.
